# Supplementary material for: An Update on Viral Hepatitis B and C in Mexico: Advances and Pitfalls in Eradication Strategies
Source: Microorganisms. 2024 Jul 3;12(7):1368. doi: 10.3390/microorganisms12071368 (PMC11279215; doi:10.3390/microorganisms12071368)
Supplement: Supplementary file 1 [file microorganisms-12-01368-s001.zip › microorganisms-3035291-supplementary.pdf]

Supplementary Material 1

Table S1. Studies analyzing the presence of HCV and HBV markers.

	n	**Hepatitis C**		**Hepatitis B**		**Identified risk factors and/or highlights**	**Ref.**
		anti-HCV+	***HCV-ARN+***	***HBsAg+***	**Other markers**		
**General population**							
Low-income population/ Guadalajara, Jalisco/ March 2016-March 2017	7 848	3.9%	ND	ND	ND	20.4% of HBsAg+ and 34.2% of anti-HCV+ had fibrosis.<br>Among HIV+ patients 5.23% were anti-HCV+ and 3.8% HBsAg+.<br>Men: women ratio was 2:1 for anti-HCV and 6:1 for HBs-Ag.<br>The age groups with the highest number of anti-HIV+ cases were 51-60 years and for HBsAg+41-50 years.<br>11.1% of anti-HCV+ in geriatrics.<br>69.9% of anti-HCV+ had abnormal liver enzymes.	[66]
	7 894	ND	ND	1.0%	ND		
**High-risk population**							
Patients of 10 hemodialysis units located in six Mexican states/ May 25, 2006-Dec. 15, 2006	368 [median age 52, range 18-93] [53.5% m., 46.5% f.]	8.4% [5.5-11.2%]	ND	7.1% [4.4-9.7%]	ND	No additional risk factors were analyzed.<br>HBV+ had been on hemodialysis longer (median time 50.5 months) or had a history of more transfusions (median 5.5 transfusions).<br>0.5% were co-infected.	[37]
Tertiary-care nurses/ Mexico City	376 [mean age 30.8±7 years] [1.86% m., 98.14% f.]	0.8%	One case positive for genotype 2b	ND	***anti-HBc+:*** 1.6%	For hepatitis C transfusions before 1992 were the main risk factor.<br>For hepatitis B the most important risk factors were tattooing and more than 4 sexual partners.	[27]
Health workers with unknown hepatitis serology in Mexico state	374 [mean age 35.5, range 18-71] [17.9% m., 82.1% f.]	1.3%	0%	0.5%	***HBV-DNA:*** 0%	The study group reported risk factors were: history of surgery (58.8%), history of work accident (15.5%), transfusions before 1992 (6.7 %), more than four sexual partners (4.2%); tattooing (2.4%), use of intravenous drugs (0.8%), intra-domiciliary contacts with patients with cirrhosis (0.6%).<br>Furthermore, 41.4% had never received hepatitis B vaccination, 58.6% reported being vaccinated (of them 1 28.8% received one dose, and 29.7% received at least two doses).	[28}
Children (<15 years old) with clinical hepatitis admitted to tertiary level hospitals/ Guadalajara, Jalisco/ 2005-2009	215 [mean age 7.2±3.8] [51.2% m., 48.8% f.]	2%	ND	3.1%	***anti-HBc+:*** 1%	History of hospitalization, surgery, and tattoos.	[68]
Multiple transfused recipients/ Mexico City	300 [mean age 30 ± 17.5] [59% m., 41% f.]	13.7%	ND	7%	ND	The main risk factors for hepatitis C were hemophilia (OR, 5.6; 2.6-12.6), more than five hospitalizations (OR, 3.8; CI 1.6-8.9), and transfusion before 1987 (OR, 19.0 95%CI, 2.0-177.7).<br>The main risk factors for hepatitis B were having end-stage renal<br>disease and being treated with hemodialysis (OR, 3.7; 95% CI, 1.4-9.9) and transplantation (OR, 4.2; 95% CI, 1.4-12.1).<br>1% were HCV/HBV coinfected.	[38]
Renal disease patients, 21 IMSS Units / Mexico State/ Jan.-Dec. 2019	1 304 [mean age 45.5±15.6] [57.5% m., 42.5% f.]. [95.8% hemodialysis, 4.2% peritoneal dialysis]	1.68%	ND	1.30%	ND	Time since renal replacement therapy was the risk factor.<br>0.15 % were coinfected.	[69]
Inmates/ Mexico City / June-Dec. 2010	17 084 [89.87% m., 10.13% f.]	3.21% (2.95-3.48)	ND	***HBsAg+ /anti-HBc+:*** 0.15% (0.10-0.22%)	***HBsAg - /anti-HBc +:*** 2.83% 2.59-3.09%)	The identified risk factors were lower educational levels, increased smoking and substance, high levels of non-sterile tattooing, physical<br>abuse and histories of sexual violence.	[67]
Inmates (10 centers)	3 232	4.8% (3.6-5.9%)	ND	0.4% (0.04-0.74%)	ND	Tattoo during incarceration, sharing tattoo materials and unprotected sex.<br>male inmates had a higher prevalence of HCV.	[70]
Injection drug users/ Tijuana and Ciudad Juárez/ ≥ 18 years old/	428 [median age 34] [91.8% m., 8.2% f.]	96.2% (92.8-98.0%)	ND	0.23%	***anti-HBc+:*** 85%	ND	[71]

Feb.-Apr.2005							
**Blood donors**							
Veracruz, Veracruz/ Jan. 1-Dec. 31, 2005	8 650	1.1%	ND	0.06%	ND	ND	[72]
Jalisco/ July 1, 2008- June 30, 2009	47 847	0.7%	17.6% (among the anti-HCV+)	0.2%	***HBV-DNA+:*** 33.3% (among HBsAg+)	ND	[73]
Guadalajara, Jalisco/ Jan. 1999-March 2005	94 806 for serological markers<br><br>100 for HCV-RNA and HBV-DNA	0.97% (0.90–1.03%)	1% (0–6%) anti-HCV-	0.23% (0.20- 0.26%	1% (0–6%)	ND	[74]
Veracruz, Veracruz/ 2006-2010	56 377	0.72% (0.63–0.76%)	ND	0. (0.08–0.14%)	ND	The risk factors for HCV were being a fisherman, living in the Papaloapan region, and having an elementary-level or lower education; and for HBsAg+ were being a cattleman and living in the Huasteca Baja region.	[75]
Puebla, Puebla	49 792 [January 2012-June 2015]	0.34% (0.28–0.38%)	0.058 (0.037–0.079)	0.10% (0.071–0.12)	***anti-HBc+: 0.79%*** (0.71–0.87%) ***HBV-DNA :*** 0.064 (0.042–0.086)	ND	[26]
	56 333 [January 2017-July 2019]	0.54% (0.48-0.60%)	0.026 (0.022–0.030)<br><br>OCI (0.005%)	1.03% (0.95–1.11)	***HBV-DNA:*** 0.044 (0.027–0.061%)		
**Coinfection**							
anti-HCV+ women/ Guadalajara, Jalisco/ >20 years old/ March 2002-June 2004	86 [mean age 53, range 21-82]	100%	63.95%	0%	***HBs-Ag-/HBV-DNA+ (OBI):*** 2.32% [3.6% in HCV-RNA+]	The main risk factors were surgeries (80%) and transfusion before 1993.<br>3.49 % were coinfected (5% in HCV-RNA+).	[76]
Inmates form 10 centers/ Guanajuato, Guanajuato /Sep 2011-Feb 2012)	3 210 [93.3% m., 6.7%f.]	4.8% (3.6-5.9%)		0.4 (0.04-0.74)		Tattoo during incarceration, sharing tattoo materials, and unprotected sex	[70]

Table S2. Studies analyzing the presence of HCV markers.

Population	n	Anti-HCV+	HCV-ARN+	Identified risk factors and/or highlights	
**General population**					
Cuernavaca, Cuernavaca/ 18-70 years old	214 [100% men)]	0.9%	ND	Exposure to sexually transmitted infections.	[77]
Lowland, Bajio Region of Mexico. May 1-Aug. 15, 2010	2 803 [mean age 38.4±13.5] [25% m., 75% f.]	1.3%	ND	History of previous transfusions (41.7% in anti-HCV+ patients).	[78]
Hidalgo/ 18-65 years old	282	0.94%	ND	The risk factors were dialysis/hemodialysis and risky sexual behavior: in a lesser proportion acupuncture, tattoo, piercing, and major surgeries before 1992.<br>The less frequently were injectable drugs use and stay in prison or jail.	[79]
Healthcare workers in Mexico City,	391 [mean age 32 years]	1.02%	0%	Among the study group, accidental puncture wounds and dental procedures were the most prevalent risk factors.	[29]

				In anti-HCV+ people, the identified risk factors were dental procedures, risky sexual behavior, accidental puncture wounds, and surgical intervention.	
Mexican living in households/ 15-49 years old /2012		0.27%	ND	Risk increased with age and was higher among those sexually active; and was lower for higher socioeconomic level	[80]
**High-risk population**					
Patients with at least one risk factor. Primary clinical care/ Mexico state/ 18-65 years old/ Nov. 2009-Nov.2010	10 524<br>47.4 ± 10.8.<br>[24.7% m., 75.3% f.]	1.2%	0.004%	Among anti-HCV+ patients the most common risk factor was blood transfusion before 1993. In the 45 patients who were HCV-RNA+, blood transfusion was the most common risk factor. None of the patients were intravenous drug users.<br>The most frequently found genotype was type 1a, with 17 cases (37.77%). The prevalence of the rest of the genotypes were: type 1b, 12 cases (26.66%); type 2a, 8 cases (17.77%); type 2b, 7 cases (15.55%); and type 3a, 1 case (2.22%).	[81]
	**Subgroup**				
	83<br>[anti-HCV+ called back patients]	100%	54.23%		
Subjects with risk factor to HCV/ ≥ 18 years/ /Nov. 2017- Sep. 2019	297 631	4.5%	3.2%	10.8% were in prison, history of acupuncture/tattooing/piercing (21%), intravenous drug use (15%) and high-risk sexual practices (12%).<br>Having at least one risk factor increased the probability of having an HCV+ result by 20% (OR = 1.20, 95% CI: 1.15-1.26). 72.9% of confirmed had at least one risk factor and 10.8% were in prison.	[82]
Inmates/ Mexico, city / 2010-2011	3 911	3.3%<br>[2.6-4.0%]	ND	43.1% among prisoners with a history of illicit drug injection similar to vitamin injection.	[83]
Inmates of 10 centers /Guanajuato State / 2011-2012	2 519	4.9%<br>(3.6±5.9%)<br>[99% of infected people were males]	ND	The risk factors were injecting drug use (OR = 7.6, 95%CI, 2.5–23.4), sharing materials for injecting-drugs (OR = 19.6, 95%CI, 4.7–<br>81.7) and being tattooed at least once before incarceration (OR = 2.1, 95%CI, 1.1–3.9), but<br>not during incarceration, were independently associated to HCV-infection.	[84]
Injection drug users/ Tijuana and Ciudad Juárez/ 2005	377<br>[All male, 31% were MSM]	96%<br>[94-99%]	ND	The risk factors were shared needles and higher numbers of lifetime female partners. Antibodies prevalence was similar independently of being MSM .	[85]
Patients and family members/ May-Sep. 2010	10 214<br>[age ranger 30-50]<br>[27% m., 73% f.]	1.17%	0.4%<br>[31.6% of anti-HCV+]	Families with cirrhosis, blood transfusion prior 1995, tattoos and piercings, and unsafe sexual practices.<br>The found genotypes were 1a (29%), 1b (48.5%), 2/2b (12.8%), and 3a (6.5%)	[86]
Patients in Health care clinics. / Jan 2006-Dec 2009	12 226 adults	1.5% (l.3-l.7%)	48.3% [among anti-HCV+]	Male gender and blood transfusion (p<0.001) were the most frequent risk factors, relatives with cirrhosis (28.3%), tattoos or piercings (25.2%), and drug users (6.9%).<br>The identified genotypes were genotype 1a (33%), 1b (21.4%), and 2a (8.5%)	[87]
Asymptomatic subjects referred for serology to HCV/ >18 years / July 2019-June 2014	7 658<br>[56.16% m.,43.84% f.]	**Global:** 4.5%<br><br>10.9%, 7.3%, 2.3% born before 1945, 1945-1965, and 1966-1992, respectively	**From 152 subjects:** 88.9%, 68.7%, and 44.4%, born before 1945, 1945-1965, and 1966-1992 respectively	ND	[88]
Asymptomatic with two or more risk factors for HCV/ Mexico City/ Feb.1-May,31-2003	300<br>[mean age 46.8±11.9]<br>[64.67% m., 35.33%]	ND	2%	The identified risk factors were manicures or pedicures with a nonpersonal instrument and more than three sexual partners	[36]
**Blood donors**					
Puebla/Jan. 2003-Dec. 2006	61 553	0.84%	ND	The main risk factors recognized in recovered donors were histories of surgery (29.1%) and of blood transfusion (6.2%), with 46 and 50% of HCV-RNA positive, respectively. Other important risk factors identified were a history of migration (15.6%), dental treatments (14.5%), multiple sex partners (5.2%), and living with infected family members (4.16%)	[89]
	**Subgroup**				
	96<br>[recovered donors]<br>[June 2005 and December 2006]	100% of donors with S/CO > 39 were HCV-RNA positive, whereas donors with lower S/CO values presented progressively reducing percentages of viral RNA detection	38.5%	The detected subtypes were: 1a (40.5%), 1b (27.0%), mixed 1a/1b (18.9%), undetermined genotype 1 (5.4%), 2a (2.7%), 2b (2.7%), and mixed 1a/2a (2.7%).<br>In 40.6% of the recovered donors the risk factor could not be identified.	

Mexico City	5 105	0.2%	90% [among anti-HCV+].	The identified genotypes were e 2b (60%) and 1a/b (40%).	[90]
**Coninfection**					
HIV+/ Monterrey/ Sept.2003-Aug.2004	140	12.1%	12.14% (94.1% patients were positive for anti-HCV antibodies)	Sexual transmission was the most frequently observed, MSM and bisexual behavior; followed by intravenous drug users. Genotype 1a was found in seven patients (41%), 1b in five patients (29.4%), 2a/c in three patients (17.6%), 2b in one patient (5.9%), and genotype 3 in the last patient (5.9%)	[91]

Table S3. Studies analyzing the presence of HBV markers.

Population	n	HBsAg+	Other markers	Identified Risk factors and/or highlights	
**General population**					
10-25 years old/ Oct. 2011-Oct. 2012	1 581	0%	***anti-HBc+/anti-HBs+ (Natural infection):*** 0.23% (0.1 a 0.52%) 0.3% (0.11–0.79%) in adolescents 0.14% (0.03–0.66%) in young adults **anti-HBc-/anti-HBs+:** (***Vaccine derived immunity):*** 44.7% (40.2-49.4%) 47.7% (42.2–53.3%) in adolescents 40.8% (33.5–48.6%) in young adults	No sociodemographic risk factors were identified.	[39]
≥ 20 years old	2 280	0.51% (0.19-2.33%) 0.54% (0.14-2.51%) in women 0.46% (0.12-1.67%) in men	ND	Sociodemographic characteristics such as sex, socioeconomic status, condom use in the first and last sexual intercourse, and age of sexual initiation were not associated with HBV infection	[41]
Puebla Medical students from a public university Age >18≤30 years/ July-Dec. 2013	201 [mean age 20.6, 20.3–20.9] [34.8% m., 65.17% f.]	0.5% [0.44–0.56%]	***anti-HBc+:*** 1% (0.87-1.13%) ***anti-HBs+:*** 47.8% (40.2-53.8%)	A history of blood transfusion/organ transplant or more than 2 sexual partners was significantly associated with anti-HBc positivity	[92]
	**Vaccine status subgroups**				
	74 vaccinated with 2 or 3 doses	0%	***anti-HBc+:*** 0% ***anti-HBs+:*** 95.9%		
	35 vaccinated with a single dose"	0%	***anti-HBc+:*** 0% ***anti-HBs+:*** 42.8%		
	30 unknown vaccination status	0%	***anti-HBc+***: 0% ***anti-HBs+:*** 23.3%		
	62 unvaccinated	1.6%	***anti-HBc+:*** 3.2% ***anti-HBs+:*** 4.8%		
Oaxaca (Mixtecos) and Michoacan (Purepechas)/ Low education and very-low income/ Jan. 2012-Dec. 2015	57 Mixtecos	10.5%	***anti-HBc+/anti-HBc+:*** 7% ***anti-HBc+:*** 5.2% ***HBV-DNA+:*** 22.8%	History of surgery, MSM, sexual workers and sexual promiscuity was the most predominant	[43]
	44 Purepechas	6.8%	***anti-HBc+/anti-HBc+:*** 0% ***anti-HBc+:*** 2.2%		

			HBV-DNA+: 2.3%		
Nahuas and Huichol Natives	306	6%	***anti-HBc+:*** 32.7%<br>***HBV-DNA+***: 16-3%<br>***anti-HBs- or anti-HBc+/-/HBV-DNA+:*** 14.2%	Transfusion, surgery, multiple sex partners	[42]
	147<br>Nahuas<br>[mean age 29±12]<br>[29.9% m., 70.1% f.]	1.4%	***anti-HBc+:*** 37%<br>***HBV-DNA+:*** 17%:		
	159<br>Huichol<br>[mean age 41±15]<br>[28.3% m., 71.7% f.]	9.4%	***anti-HBc+:*** 28%<br>HBV-DNA+:16%		
Puebla/2003-2009	120 552	0.066% (0.053-0.079%)	***anti-HBC+:*** 1.19% (1.12 - 1.26%)<br>***anti-HBc+/HBsAg-:*** 17.3% (11.5 - 23.1%).	All HBV-DNA+ were genotype H.<br>"a" determinant mutations found were R122K, P127L, Y134F.	[64]
	156<br>[2009]<br>(anti-HBc+/HBsAg-)	0%	***HBV-DNA+:*** 17.3% (11.5 - 23.1%) [		
**High-risk-population**					
Psychiatric patients/ Durango	99<br>[mean age 39.4, range 18-80]<br>[75% m., 25% f.]	7.1%	***anti-HBs+:*** 5.1%	Age of 45 years and older were associated (OR=4.27; 95%CI, 1.02-18.78)	[93]
**Blood donors**					
Mérida, Yucatán/ Jan. 2002-Dec. 2004	39 933	0.2%	ND	ND	[94]
**Coinfections**					
HIV positive/HBs-Ag-/2009-2011	49<br>[mean age 45, range 18-73]	0%	***Anti-HBc+:*** 2.6%<br>***HBV-DNA+:*** 49%	HIV viral load (OR 8.75; 95% CI: 2.26-33-79).	[95]
HIV+/ West of Mexico / Jan.-Dec. 2002	38<br>[mean age 36, range 22–70]<br>[84.2% m, 5.8% f.]	7.9%	***HBs-Ag-/HBV-DNA+ (OBI):*** 18.4 %<br>***anti-HBs+/IgG anti-HBC+/HBs-Ag-:*** 10%<br>***anti-HBc+:*** 20%	Additional risk factors were not statistically significant<br>26.3% were HIV/HBVcoinfected.	[96]
